# Rutin Stabilises β‐Catenin Through GSK3β Inhibition to Promote Hair Follicle Regeneration

**DOI:** 10.1111/cpr.70185

**Published:** 2026-02-23

**Authors:** Yanyan Zhang, Yuanjie Zhu, Mengyu Jin, Jing Chen, Siqi Yuan, Minjia Yuan, Yuou Sha, Qingmei Liu, Wenyu Wu, Juan Wang, Xiaolei Ding

**Affiliations:** ^1^ Institute of Geriatrics (Shanghai University), Affiliated Nantong Hospital of Shanghai University (The Sixth People's Hospital of Nantong), School of Medicine, Shanghai University Nantong China; ^2^ Joint International Research Laboratory of Biomaterials and Biotechnology in Organ Repair (Ministry of Education) Shanghai University Shanghai China; ^3^ Shanghai Engineering Research Center of Organ Repair, School of Medicine Shanghai University Shanghai China; ^4^ Department of Dermatology, Naval Medical Centre Naval Medical University Shanghai China; ^5^ Shanghai Qiran Biotechnology Co. Ltd. Shanghai China; ^6^ Department of Dermatology, Huashan Hospital, Shanghai Institute of Dermatology Fudan University Shanghai China

## Abstract

Rutin directly binds to GSK3β, stabilising β‐catenin and activating Wnt/β‐catenin signalling to drive hair cycle.

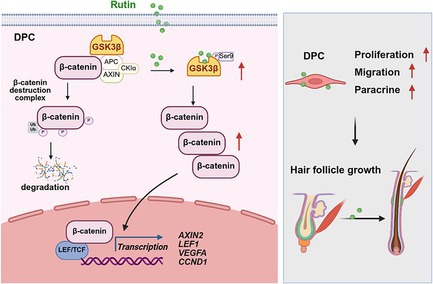

To the Editor,

1

Hair follicle (HF) regeneration depends on a tightly coordinated cyclic process consisting of three distinct phases: telogen (resting), anagen (growth) and catagen (regression) [1]. Disruption of the HF cycling by stress, hormones, ageing and pharmacological agents frequently results in hair loss [2], which affects approximately 30% of the global population [3]. Current therapies for hair loss, including minoxidil, finasteride and surgical transplantation, offer only limited benefits and are often associated with adverse effects or procedural challenges [4, 5], highlighting the need for safe, mechanism‐based agents that can reactivate dormant follicles and sustain hair growth.

Wnt/β‐catenin signalling axis plays a central role in HF morphogenesis and cycle, a process primarily orchestrated by the dermal papilla cells (DPCs). As the key signalling centre within the follicle bulb, Wnt activation in the DP enhances its intrinsic inductivity to drive the transition into the anagen growth phase [6]. Glycogen synthase kinase 3β (GSK3β) is a key regulator of this pathway. It targets and phosphorylates β‐catenin, thereby triggering its ubiquitination and subsequent degradation via the proteasome [7]. Crucially, GSK3β activity is modulated through site‐specific phosphorylation [8]. For example, phosphorylation at serine 9 (Ser9) renders GSK3β catalytically inactive, blocking β‐catenin phosphorylation, allowing its nuclear accumulation and activating downstream targets, including *VEGFA* and *Cyclin D1* [9].

Rutin, a naturally occurring flavonol glycoside abundant in many plants, is known for vascular‐protective, antioxidant and anti‐inflammatory effects and has been used clinically with an excellent safety profile [10, 11, 12]. Although rutin has been reported to modulate Wnt signalling in other contexts [13], its specific role in hair growth and the precise mechanism underlying its modulation of Wnt signalling remain unknown. Here, we investigated the effects of rutin on hair growth and elucidated the molecular mechanism by which it activates Wnt/β‐catenin signalling.

To evaluate in vitro effects of rutin, we isolated primary DPCs. CCK8 and Ki67 immunofluorescence staining showed that rutin promoted DPC proliferation, with its effect being superior to that of minoxidil (Figure S1A–C). Scratch assays revealed enhanced DPC migration compared with controls (Figure S1D). Quantitative real‐time polymerase chain reaction (RT‐qPCR) analysis further showed that rutin upregulated *Versican*, *FGF7*, *PCNA* and *VEGFA* expression (Figure S1E–H), genes encoding well‐characterised DPC‐derived mediators of the hair cycle. Together, these findings suggest that rutin activates DPCs and increases the production of growth factors.

To validate these findings in a physiologically relevant system, we performed ex vivo HF organ cultures. Human anagen HFs were isolated as previously described [14] (Figure S2A,B), and subsequently cultured in basic medium supplemented with either vehicle, minoxidil, or rutin. Images were captured on days 0, 3 and 6 to monitor follicle morphology and HF elongation (Figure 1A). In the ex vivo system, cultured follicles continue to grow transiently before entering catagen, characterised by DPC detachment from the hair matrix and hair bulb regression. Morphological changes and alterations in HF length thus serve as key indicators of follicular activity. Notably, rutin induced a dose‐dependent elongation of the HFs, with a significant effect observed at 50 μM, exceeding that of minoxidil (Figure 1A). In line with this, rutin treatment increased the number of Ki67^+^ DPCs and hair matrix cells, indicating enhanced cellular proliferation within the HF compartment (Figure 1B). These findings demonstrate that rutin promotes HF elongation and stimulates follicular proliferation in an ex vivo setting, supporting its capacity to sustain hair growth and delay catagen onset.

**FIGURE 1:**
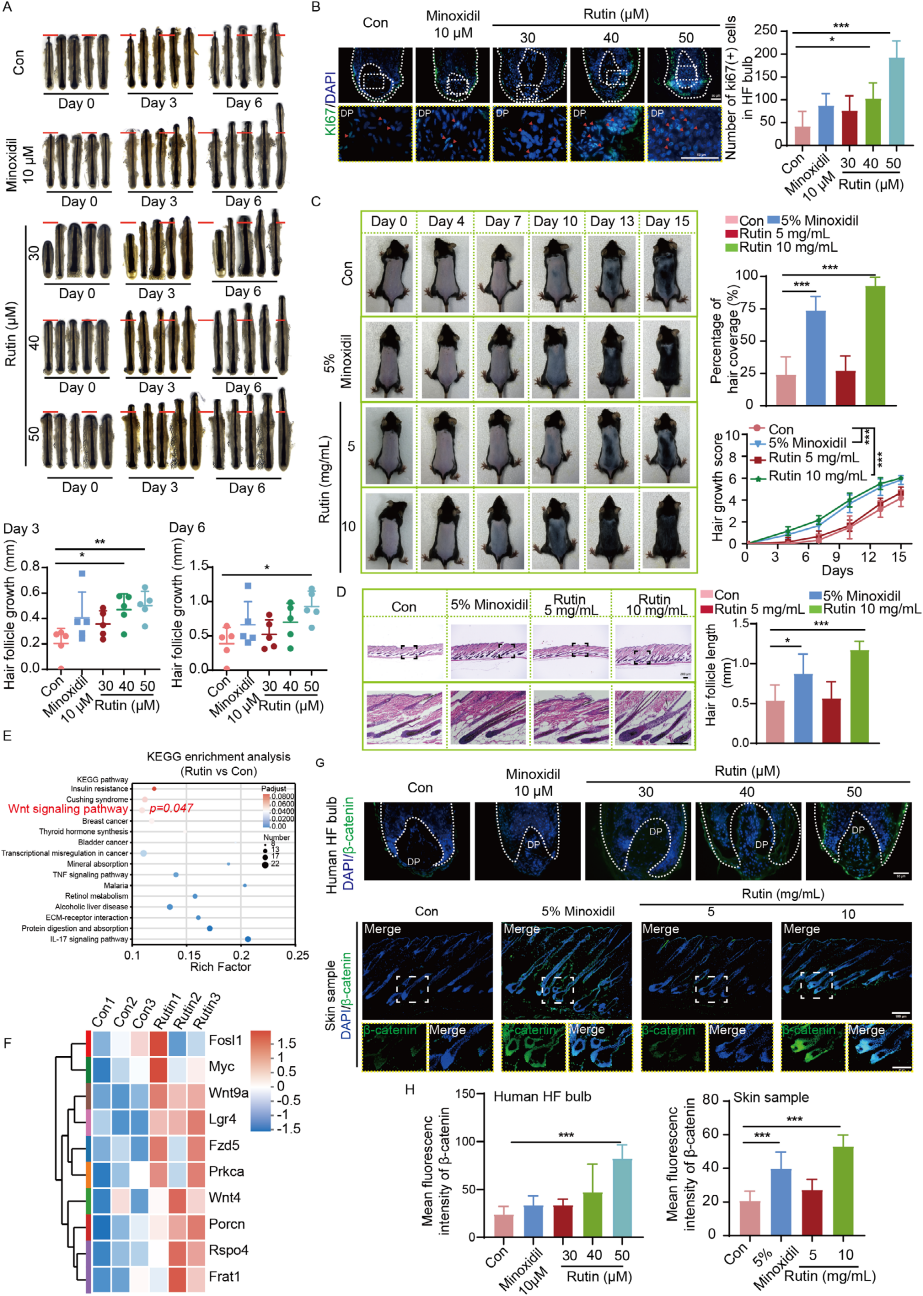
Rutin promotes hair growth and activates β‐catenin signalling pathway. (A) Images and quantification of hair shaft length from human hair follicles (HFs) treated with rutin or minoxidil (*n* = 5, one‐way ANOVA, followed by Tukey's multiple comparisons test). (B) Ki67 immunofluorescence staining showing cell proliferation of HFs after exposure to rutin or minoxidil at day 6 (*n* = 5, one‐way ANOVA, followed by Tukey's multiple comparisons test). (C) Representative images and quantification of mouse hair regrowth. C57BL/6 mice were treated topically once a day with rutin (5 and 10 mg/mL), minoxidil (5%) or vehicle control (65% ethanol), and were imaged three times a week (*n* = 6, one‐way ANOVA and two‐way ANOVA, followed by Tukey's multiple comparisons test). (D) Vertical images of H&E staining of mouse skin and corresponding quantification of HF length on day 15 (*n* = 6, one‐way ANOVA, followed by Tukey's multiple comparisons test). Scale bar = 200 μm. (E) KEGG analysis of the DEGs. (F) Heatmap of Wnt signalling pathway associated genes. (G, H) β‐catenin immunofluorescence staining of human HFs (G, *n* = 5) and mouse skin (H, *n* = 6, one‐way ANOVA, followed by Tukey's multiple comparisons test). *p < 0.05, **p < 0.01 and ***p < 0.001.

We next evaluated the in vivo effects of rutin using a depilation‐induced hair regeneration model in mice. Following synchronised HF entry into telogen by depilation, mice were treated topically once daily with vehicle, rutin (5 or 10 mg/mL) or 5% minoxidil, the clinical gold standard for hair loss. Hair regrowth was monitored and photographed on days 0, 4, 7, 10, 13 and 15 (Figure 1C). As early as day 7, dorsal skin in both the high‐dose rutin (10 mg/mL) and minoxidil groups began to exhibit grey pigmentation, an established visual marker of anagen induction, whereas the control and low‐dose rutin groups retained pink skin, indicative of a persistent telogen state. By day 15, high‐dose rutin and minoxidil‐treated mice displayed near‐complete hair coverage (> 80%), significantly surpassing the regrowth observed in vehicle‐treated controls (∼25%). Quantitative hair growth scoring confirmed a dose‐dependent effect, with high‐dose rutin achieving results comparable to minoxidil (Figure 1C). Histological analysis revealed the marked increases in HF length and skin thickness in the 10 mg/mL rutin and minoxidil groups relative to controls (Figure 1D, Figure S3A), indicative of anagen progression. Additionally, both groups exhibited an increased number of active follicles per unit area (Figure S3A), suggesting enhanced follicular activation. The Ki67 immunofluorescence staining also showed that both 10 mg/mL rutin and minoxidil treatments substantially increased the number of Ki67^+^ proliferative cells within the DP, hair matrix and outer root sheath (Figure S3B), consistent with enhanced anagen induction. Similarly, hair growth associated genes, including *Versican*, *Fgf7* and *Vegfα* were also upregulated by rutin (Figure S4), further supporting its ability to facilitate follicular growth programmes.

Transcriptomic profiling of skin tissues collected on day 15 post‐treatment identified 1270 differentially expressed genes (665 upregulated, 605 downregulated) following rutin exposure (Figure S5A,B). Kyoto Encyclopedia of Genes and Genomes (KEGG) analysis revealed that these DEGs tightly focused on the Wnt signalling pathway (Figure 1E). GSEA analysis confirmed the upregulation of the Wnt signalling pathway after rutin treatment (Figure S5C). Specifically, multiple Wnt/β‐catenin pathway target genes and its components were upregulated after rutin treatment (Figure 1F), indicating that the Wnt/β‐catenin signalling pathway may be involved in rutin‐mediated therapeutic effects. To confirm this, we conducted immunofluorescence staining. Results from both HF organs and skin tissues showed that rutin markedly elevated β‐catenin levels within the epidermis and follicular epithelium, showing a 3.5‐fold increase in HFs and a 2.5‐fold increase in skin compared to control (Figure 1G,H, Figure S6).

To define the mechanism by which rutin activates Wnt/β‐catenin signalling, we examined β‐catenin expression dynamics. Our results revealed that rutin increased the β‐catenin protein levels in a dose‐ and time‐dependent manner (Figure 2A, Figure S7A), while it had no effect on the transcription of *CTNNB1*, which encodes β‐catenin (Figure 2B), suggesting post‐transcriptional regulation. To determine whether rutin affects β‐catenin protein stability, we treated DPCs with the protein synthesis inhibitor cycloheximide (CHX) with or without rutin. Figure 2C showed that rutin treatment delayed β‐catenin degradation in the presence of CHX, indicating an extended protein half‐life. These data suggest that rutin stabilises β‐catenin by interfering with its proteasomal turnover. Since nuclear accumulation of β‐catenin is essential for activating downstream target genes, we next examined its subcellular distribution. Immunofluorescence analysis demonstrated increased β‐catenin levels in both the cytoplasm and nucleus of DPCs treated with rutin (Figure S7B). Consistently, rutin upregulated the expression of canonical Wnt/β‐catenin target genes, including *CCND1*, *AXIN2* and *LEF1*, as measured by RT‐qPCR (Figure S7C–E). Taken together, these findings indicate that rutin promotes β‐catenin stabilisation, enhances its nuclear translocation and facilitates transcriptional activation of downstream effectors, thereby augmenting Wnt/β‐catenin signalling.

**FIGURE 2:**
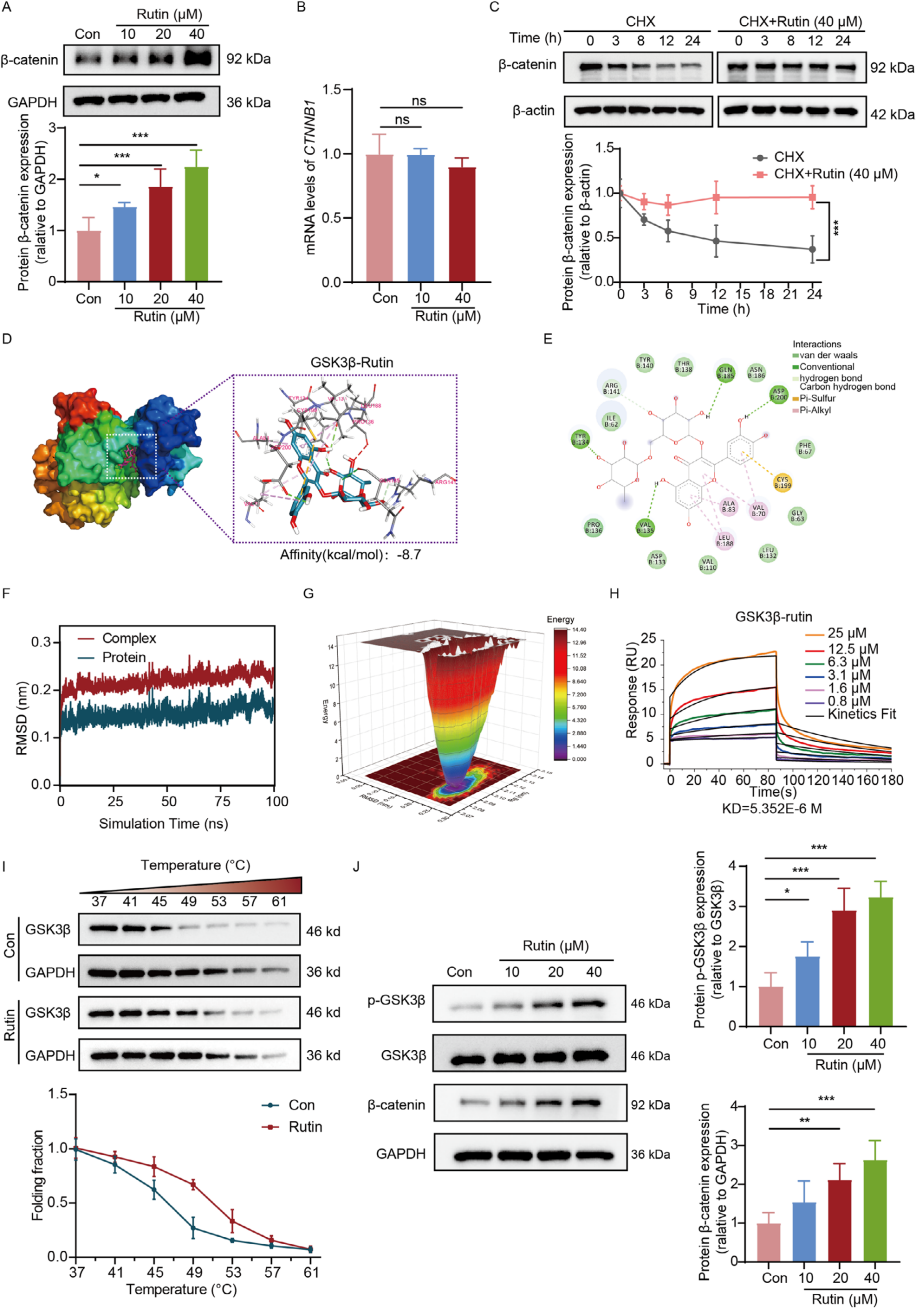
Rutin directly targets GSK3β to activate Wnt/β‐catenin signalling pathway. (A, B) DPCs were treated with various concentrations of rutin for 24 h. (A) Western blot analysis of β‐catenin protein level (*n* = 5, one‐way ANOVA, followed by Tukey's multiple comparisons test). (B) RT‐qPCR analysis of *CTNNB1 mRNA* expression (*n* = 3, one‐way ANOVA, followed by Tukey's multiple comparisons test). (C) Western blot analysis of β‐catenin protein level in DPCs in the presence of CHX with or without rutin (40 μM) as indicated (*n* = 5, two‐way ANOVA, followed by Tukey's multiple comparisons test). (D, E) Molecular docking illustrating the binding mode of rutin and GSK3β. (F, G) Molecular dynamics simulation illustrating the stability binding of rutin and GSK3β via RMSD (F) and FEL (G). (H) SPR detecting the binding affinity of rutin and GSK3β. (I) Representative images of CETSA and quantification of GSK3β protein expression (*n* = 4, two‐way ANOVA, followed by Tukey's multiple comparisons test). (J) Western blot analysis of p‐GSK3β, GSK3β and β‐catenin protein levels in DPCs after treatment with rutin for 24 h. GAPDH served as the loading control (*n* = 5, one‐way ANOVA, followed by Tukey's multiple comparisons test). *p < 0.05, **p < 0.01 and ***p < 0.001.

To further dissect the molecular mechanism by which rutin stabilises β‐catenin, we first examined the possibility of direct interaction between rutin and β‐catenin. Molecular docking predicted a modest binding affinity of −5.5 kcal/mol between rutin and β‐catenin (Figure S8A), suggesting limited direct interaction. We therefore hypothesised that rutin may regulate β‐catenin turnover indirectly via upstream modulators. Among these, GSK3β is a key regulator of β‐catenin stability [7], we therefore studied whether rutin modulates β‐catenin through targeting GSK3β. Molecular docking analysis revealed a binding affinity of approximately −8.7 kcal/mol, indicative of strong binding affinity (Figure 2D). In addition, rutin interacted with GSK3β via forming four hydrogen bonds with residues: TYR‐134, VAL‐135, GLN‐185, ASP‐200, van der Waals as well as one carbon hydrogen bond (Figure 2E). Moreover, molecular dynamic simulations were conducted to test the stability of the rutin‐GSK‐3β complex. The root mean square deviation (RMSD) and radius of gyration (Rg) trajectory indicated that the rutin‐GSK3β complex maintained relatively stable values (Figure 2F, Figure S9A), suggesting that the system maintained a stable and compact state. RMSF analysis of equilibrated trajectories revealed high structural flexibility in residues 290–300 within the rutin‐GSK3β binding pocket (Figure S9B). Molecular mechanics‐Poisson Boltzmann surface area (MM‐PBSA) was used to calculate the binding free energy in the rutin‐GSK3β complex. This system exhibited lower energy levels with a binding energy of approximately −81.36 kJ/mol (Figure S9C). The individual contributions of various energy were listed in Table S1. Of note, van der Waals interactions predominated in the molecular recognition of GSK3β, indicating their central role in molecular recognition. The distribution of free energy across different conformational states of this system was analysed using the free energy landscape (FEL). The FEL landscape revealed convergence of the rutin‐GSK3β complex towards the dominant low‐energy base (Figure 2G). To further confirm the direct interaction between rutin and GSK3β, surface plasmon resonance (SPR) was performed. As shown in Figure 2H, rutin bound to GSK3β with an equilibrium dissociation constant (*K*_D_) of 5.352 μM, indicating a strong binding interaction. Rutin dissociated immediately from GSK3β after flow termination, indicating noncovalent interactions. Meanwhile, CETSA analysis revealed that rutin potentially enhanced the thermal stability of GSK3β as the temperature increased in DP cells (Figure 2I), consolidating the binding interaction. Functionally, we found that rutin treatment significantly increased the inhibitory phosphorylation of GSK3β at Ser9, without affecting total protein levels (Figure 2J). This suggests that rutin inactivates GSK3β to promote β‐catenin stabilisation.

Given prior evidence that AKT activation can mediate GSK3β Ser9 phosphorylation and that rutin may inactivate GSK3β by activating AKT [15], we further investigated whether AKT signalling contributes to this process. Molecular docking predicted a potential rutin‐AKT interaction (Figure S10A). Accordingly, we tested AKT's role. Rutin indeed enhanced AKT phosphorylation, an effect abolished by AKT inhibitor MK‐2206 (Figure S10B,C). However, MK‐2206 failed to attenuate rutin‐induced GSK3β phosphorylation (Figure S10D), indicating that AKT activation was not the main contribution for rutin's action on inhibiting GSK3β and the consequent β‐catenin stabilisation.

To further confirm that rutin promoted β‐catenin stabilisation and consequent DP activity via targeting GSK3β, we knockdown GSK3β by siRNA (Figure S11A). We found that knockdown of GSK3β significantly dampened the ability of rutin to promote DPC proliferation, β‐catenin protein and its target gene *CCND1* expression in DPCs (Figure S11B–D). This result indicates that GSK3β plays a key role in mediating the biological effects of rutin. Meanwhile, we did note, however, that in GSK3β‐knockdown cells, rutin still retained a residual degree of activity. This observation may be consistent with the multi‐target nature of many natural compounds [16]. It is possible that additional pathways partially contribute to the overall effects. Collectively, these results demonstrate that rutin promotes β‐catenin signalling primarily through direct inhibition of GSK3β.

In conclusion, we identify rutin as a previously unrecognised modulator of HF dynamics. Specifically, the topical administration of rutin significantly accelerated anagen induction and promoted hair shaft elongation in vivo and ex vivo. Mechanistically, we demonstrated that rutin enhanced Wnt/β‐catenin signalling by stabilising β‐catenin protein levels through direct inhibition of GSK3β activity. Notably, rutin is already approved as an adjunct therapy for hypertension and possesses well‐established pharmacokinetics and safety profiles [17], enabling rapid translational potential. Moving forward, further studies to investigate and compare the effects of other GSK3β modulators, such as Lithium, a clinically available drug in hair loss associated diseases would be interesting.

Our findings provide mechanistic insight into the pro‐regenerative properties of rutin and support its development as a safe and effective therapeutic for alopecia and related hair disorders.

## Author Contributions

X.D., J.W. and W.W. conceived and supervised the project, wrote the manuscript and reviewed the writing. Ya.Z. and Yu.Z. conducted experiments, performed statistical analysis and made the figures. All authors contributed to this manuscript and approved the submitted version.

## Funding

This work was supported by the National Natural Science Foundation of China (82204450 and 82272278), National Key Research and Development Program of China (2023YFC2509000) and Shanghai Qiran Biotechnology Co. Ltd.

## Conflicts of Interest

The authors declare no conflicts of interest.

## Supporting information


**Figure S1:** Rutin activates DPCs. (A) Chemical structure of rutin. (B) CCK8 analysis of cell viability of DPCs after exposure to various concentrations of rutin for 24 h (*n* = 4 One‐way ANOVA, followed by Tukey's multiple comparisons test). (C) Ki67 immunofluorescence staining showing cell proliferation of DPCs after exposure to rutin (40 μM) or minoxidil (10 μM) for 24 h (*n* = 3, one‐way ANOVA, followed by Tukey's multiple comparisons test). (D) Scratch assay analysis of cell migration of DPCs after treatment with rutin (*n* = 4, unpaired Student's *t*‐test). (E–H) RT‐qPCR analysis of hair growth associated genes in DPCs after exposure to rutin (40 μM) or minoxidil (10 μM) for 24 h (*n* = 3, one‐way ANOVA, followed by Tukey's multiple comparisons test). Data are mean ± SD. *p < 0.05, **p < 0.01 and ***p < 0.001.
**Figure S2:** (A) The isolation of single hair follicles from human occipital scalp region. (B) Representative images showing hair follicle morphology in anagen and catagen, showing DPCs detachment from the hair matrix during catagen.
**Figure S3:** Rutin promotes hair growth in vivo. (A) Transverse images of H&E staining of mouse skin on day 15. (A, right) Quantification of skin thickness and number of HFs in transverse sections (*n* = 6, one‐way ANOVA, followed by Tukey's multiple comparisons test). Scale bar = 200 μm. (B) Ki67 immunofluorescence staining showing cell proliferation of mouse skin on day 15. *p < 0.05, ***p < 0.001.
**Figure S4:** Rutin promotes the expression of genes involved in hair growth in vivo. RT‐qPCR analysis of *Versican*, *Fgf7*, *Vegfa* mRNA expression in mouse skin on day 15 post‐administration (*n* = 5, one‐way ANOVA, followed by Tukey's multiple comparisons test). Data are mean ± SD. **p < 0.01 and ***p < 0.001.
**Figure S5:** Transcriptomic analysis of the gene profiling of skin tissues after rutin treatment. (A) The number of DEGs of RNA sequencing results of mouse skin on day 15 (*n* = 3). (B) Volcano plot illustrating the expression patterns of DEGs for mouse skin after topical administration of rutin (10 mg/mL). (C) GSEA illustrating Wnt signalling pathway enriched in mouse skin.
**Figure S6:** Higher‐magnification views of Figure 1G.
**Figure S7:** Rutin activates β‐catenin signal in vitro. (A) Western blot analysis of β‐catenin protein level in DPCs after exposure to rutin (40 μM) for indicated time. *n* = 5. (B) β‐catenin immunofluorescence staining and (C–E) RT‐qPCR analysis of *CCND1*, *AXIN2* and *LEF1* genes in DPCs following treatment with rutin (10, 20, 40 μM) for 24 h (*n* = 3, one‐way ANOVA, followed by Tukey's multiple comparisons test). Data are mean ± SD. *p < 0.05, **p < 0.01 and ***p < 0.001.
**Figure S8:** Molecular docking analysis of rutin/β‐catenin complex.
**Figure S9:** Molecular dynamic simulations analysis of rutin/GSK3β complex. (A–C) Molecular dynamics simulation illustrating the stability binding of rutin and GSK3β via Rg (A), RMSF (B) and energy (C).
**Figure S10:** AKT is not required for GSK3b phosphorylation. (A) Molecular interactions between rutin and AKT (A). (B–D) Western blot analysis of AKT, p‐AKT, GSK3β, p‐GSK3β and β‐catenin protein levels in DPCs with or without AKT inhibitor MK‐2206 treatment in the presence of rutin for 24 h. *n* = 5 for B and C. *n* = 3 and *n* = 4 for D. Data are mean ± SD. *p < 0.05, **p < 0.01 and ***p < 0.001, one‐way ANOVA, followed by Tukey's multiple comparisons test.
**Figure S11:** GSK3β is essential for rutin functions in DPCs. (A) Western blot analysis to detect GSK3β protein expression after transfected with GSK3β siRNAs. (B) CCK‐8 analysis of DPC proliferation in the presence or absence of rutin treatment after GSK3β knockdown (*n* = 4). (C) Western blot analysis of β‐catenin protein EXPRESSION in DPCs in the presence or absence of rutin treatment after GSK3β knockdown (*n* = 5). (D) RT‐qPCR analysis of *CCND1* mRNA level in DPCs in the presence or absence of rutin treatment after GSK3β knockdown (*n* = 3). Data are mean ± SD. *p < 0.05, ***p < 0.001, one‐way ANOVA, followed by Tukey's multiple comparisons test.
**Table S1:** Binding energy and composition in a stable state (unit: kJ/mol).

## Data Availability

The data that support the findings of this study are available from the corresponding author upon reasonable request.
