# Supplementary material for: Rutin Stabilises β‐Catenin Through GSK3β Inhibition to Promote Hair Follicle Regeneration
Source: Cell Prolif. 2026 Feb 23;59(4):e70185. doi: 10.1111/cpr.70185 (PMC13052294; doi:10.1111/cpr.70185)
Supplement: Supplementary file 1 — Figure S1: Rutin activates DPCs. (A) Chemical structure of rutin. (B) CCK8 analysis of cell viability of DPCs after exposure to various concentrations of rutin for 24 h (n = 4 One‐way ANOVA, followed by Tukey's multiple comparisons test). (C) Ki67 immunofluorescence staining showing cell proliferation of DPCs after exposure to rutin (40 μM) or minoxidil (10 μM) for 24 h (n = 3, one‐way ANOVA, followed by Tukey's multiple comparisons test). (D) Scratch assay analysis of cell migration of DPCs after treatment with rutin (n = 4, unpaired Student's t‐test). (E–H) RT‐qPCR analysis of hair growth associated genes in DPCs after exposure to rutin (40 μM) or minoxidil (10 μM) for 24 h (n = 3, one‐way ANOVA, followed by Tukey's multiple comparisons test). Data are mean ± SD. *p < 0.05, **p < 0.01 and ***p < 0.001. Figure S2: (A) The isolation of single hair follicles from human occipital scalp region. (B) Representative images showing hair follicle morphology in anagen and catagen, showing DPCs detachment from the hair matrix during catagen. Figure S3: Rutin promotes hair growth in vivo. (A) Transverse images of H&E staining of mouse skin on day 15. (A, right) Quantification of skin thickness and number of HFs in transverse sections (n = 6, one‐way ANOVA, followed by Tukey's multiple comparisons test). Scale bar = 200 μm. (B) Ki67 immunofluorescence staining showing cell proliferation of mouse skin on day 15. *p < 0.05, ***p < 0.001. Figure S4: Rutin promotes the expression of genes involved in hair growth in vivo. RT‐qPCR analysis of Versican, Fgf7, Vegfa mRNA expression in mouse skin on day 15 post‐administration (n = 5, one‐way ANOVA, followed by Tukey's multiple comparisons test). Data are mean ± SD. **p < 0.01 and ***p < 0.001. Figure S5: Transcriptomic analysis of the gene profiling of skin tissues after rutin treatment. (A) The number of DEGs of RNA sequencing results of mouse skin on day 15 (n = 3). (B) Volcano plot illustrating the expression patterns of DEGs for mou [file CPR-59-e70185-s001.docx]

**Rutin stabilizes β-catenin through GSK3β inhibition to promote hair follicle regeneration**

Yanyan Zhang^1,2,3*^, Yuanjie Zhu^4*^, Mengyu Jin^1,2,3^, Jing Chen^1,2,3^, Siqi Yuan^1,2,3^, Minjia Yuan^5^, Yuou Sha^6^, Qingmei Liu^6^, Wenyu Wu^6#^, Juan Wang^1,2,3#^, Xiaolei Ding^1,2,3#^

1 Institute of Geriatrics (Shanghai University), Affiliated Nantong Hospital of Shanghai University (The Sixth People’s Hospital of Nantong), School of Medicine, Shanghai University, Nantong, 226011, China

2 Joint International Research Laboratory of Biomaterials and Biotechnology in Organ Repair (Ministry of Education), Shanghai University, Shanghai 200444, China

3 Shanghai Engineering Research Center of Organ Repair, School of Medicine, Shanghai University, Shanghai 200444, China

4 Department of Dermatology, Naval Medical Centre, Naval Medical University, Shanghai, 200052, China

5 Shanghai Qiran Biotechnology Co., Ltd., Shanghai 201702, China

6 Department of Dermatology, Huashan Hospital, Shanghai Institute of Dermatology, Fudan University, Shanghai 200040, China

* These authors contributed equally to this article.

#Corresponding authors: Dr. Wenyu Wu, No. 12, Urumqi Middle Road, Jing’an District, Shanghai, China. Email: [wuwenyu@huashan.org.cn](mailto:wuwenyu@huashan.org.cn). Tel.: +86-021-52889999. Fax.: +86-021-62489191; Dr. Juan Wang, No. 99, Shangda Road, Baoshan District, Shanghai, China. Email: juanw@shu.edu.cn. Tel.: +86-181-1635-1753. Fax.: +86-021-66131156; Dr. Xiaolei Ding, No. 99, Shangda Road, Baoshan District, Shanghai, China. Email: xlding@shu.edu.cn. Tel.: +86-137-6457-1130. Fax.: +86-021-66131156.

**Materials and methods**

**Cells and reagents**

Human DPCs were isolated from anagen HFs by mechanical dissociation, following a previously described protocol[1]. The DPCs were cultured in DMEM supplemented with 10% fetal bovine serum (FBS, Gibco) and 1% penicillin/streptomycin (Thermo Fisher). Cells were maintained at 37°C in a humidified atmosphere of 5% CO₂, with medium changed every 2-3 days. DPCs with passage numbers less than 5 were used in this study.

Rutin (Cat#S2350), MK-2206 2HCl (Cat#S1078), minoxidil (Cat#S1383), and cycloheximide (Cat#S7418) were purchased from Selleck Chemicals (Houston, TX, USA).

**Cell viability**

Cell viability was determined using Cell Counting Kit-8 (CCK-8, Meilunbio) according to the manufacturer's protocol. Briefly, DPCs were seeded in 96-well plates at a density of 5×10³ cells/well and allowed to adhere for 24 hours. Next, DPCs were treated with various concentrations of rutin (1, 5, 10, 20, 30, 40 μM). After 24 hours of treatment, 10 μL of CCK-8 reagent was added to each well and incubated for another 1 hour at 37°C. Absorbance was measured at 450 nm using a microplate reader (Infinite e plex, Tecan, Austria).

**Scratch assay**

The scratch wound healing was performed to evaluate the migration ability of DPCs. DPCs were seeded into 24-well plates at a density of 5×10^4^ cells per well, and a sterile pipette tip was used to make a straight line in the center of the well. Following PBS washing to remove debris, cells were maintained in starvation medium containing 1% FBS with or without rutin treatment. The scratch closure images were captured at 0 and 36 hours using a microscope. Image J software was used to measure closure rate of the scratch area.

**Quantitative real-time polymerase chain reaction (RT-qPCR)**

Total RNA was extracted using RNA extraction kits (Vazyme). RNA concentrations were determined by spectrophotometry. cDNA was synthesized using reverse transcription kits (Vazyme), followed by quantitative PCR with SYBR Green Master Mix (Vazyme). Gene expression was analyzed via the 2^-ΔΔCt^ method and normalized against *GAPDH* mRNA. All experiments were performed in triplicate. Primers are listed as follows.

| **Name** | **Forward (5’-3’)** | **Reverse (5’-3’)** |
| --- | --- | --- |
| Human *VERSSICAN* | AGGTGGTCTACTTGGGGTGA | GTGATGCAGTTTCTGCGAGG |
| Human *FGF7* | AAGTTGCACCAGGCAGACAA | AGTTCAGTTGCTGTGACGCT |
| Human *VEGFA* | TGTCTAATGCCCTGGAGCCT | TAACTCAAGCTGCCTCGCC |
| Human *PCNA* | GCCCTGGTTCTGGAGGTAAC | TAGCTGGTTTCGGCTTCAGG |
| Human *CCND1* | GCTGCGAAGTGGAAACCATC | CCTCCTTCTGCACACATTTGAA |
| Human *AXIN2* | TGGCTATCTCCCCACCTTGA | CAGTTTCCGTGGACCTCACA |
| Human *LEF1* | TTCTTGGCAGAAGGTGGCAT | GCAGCTGTCATTCTTGGACC |
| Human *CTNNB1* | GAAGGTCTGAGGAGCAGCTT | GCCATTGTCCACGCTGGATT |
| Human *GAPDH* | CGGAGTCAACGGATTTGGTC | GACAAGCTTCCCGTTCTCAG |
| Mouse *Versican* | ACAATAGGATGGCTCTGAA | TAGTAACCAGGCAACAGT |
| Mouse *Fgf7* | TGGGCACTATATCTCTAGCTTGC | GGGTGCGACAGAACAGTCT |
| Mouse *Vegfα* | CCTGGCCCTCAAGTACACCTT | TCCGTACGACGCATTTCTAG |
| Mouse *Gapdh* | CATGTTTGTGATGGGTGTGA | AATGCCAAAGTTGTCATGGA |

**Isolation and culture of human HFs**

This study was conducted following the principles of the Declaration of Helsinki. Human anagen HFs were obtained from the occipital scalp region during routine hair transplantation procedures with written consent from patients and approval from the ethics committee (approval No. 2024M-001). Freshly extracted HFs were washed thrice with ice-cold PBS, then microdissected under a stereomicroscope (Leica MZ8/Olympus) to remove excess adipose and connective tissue. Isolated follicles were cultured in 24-well plates containing Williams E medium (Gibco) supplemented with 10 μg/mL insulin, 2 mM L-glutamine, 10 ng/mL hydrocortisone, and 100 U/mL penicillin/streptomycin, maintained at 37°C with 5% CO₂. After 24 hours of stabilization, follicles exhibiting anagen characteristics were selected for experiments, with medium changed every 3 days. The HFs were treated with various concentrations of rutin or minoxidil (10 μM) and photographed with via a Leitz Labovert inverted microscope (Leitz Labovert FS). Image J was used to calculate the relative length.

**Animal experiments**

​​All animal experiments were conducted in accordance with the Institutional Animal Care and Use Committee of Shanghai University approval. Male 7-week-old C57BL/6 mice purchased from Shanghai Model Organisms (Shanghai, China) were used in this study. After one-week acclimation, mouse dorsal hair was shaved using electric clippers followed by depilatory cream to synchronize HFs. Animals were randomly divided into 4 groups (n=6 for each group): vehicle control (65% ethanol), rutin (10 mg/mL in 65% ethanol), rutin (5 mg/mL in 65% ethanol) and minoxidil (5% in 65% ethanol) as positive control. Treatments were administered topically (200 μL) once daily for 15 days. Hair regrowth was monitored by digital photography on days 0, 4, 7,10, 13 and 15. At the end of the experiment, skin samples were collected for subsequent study. Image J software was used to analyze the percentage of hair coverage. Hair growth score was quantified based on 0-6 scoring system [2], which reflects hair cycle progression through established skin color changes, where pink skin=1 point, light pink/pinkish white skin=2 points, pure white skin=3 points, off-white/grayish white skin=4 points, gray skin=5 points, and dark gray skin=6 points.

**Histological analysis**

Skin tissues were fixed with 4% paraformaldehyde (PFA) and paraffin embedded. Subsequently, tissues were longitudinally or transversely sectioned (5 μm). H&E staining was performed according to standard protocols [3]. Stained sections were imaged using light microscopy. Image J software was used to measure the length and number of HFs as well as skin thickness.

**Immunofluorescence analysis**

HFs and skin tissues were embedded in optimal cutting temperature compound (OCT) and sectioned at thickness of 5 μm. Sections were blocked with goat serum for 1 hour at room temperature. Then, primary antibodies were applied to the sections and incubated overnight at 4°C. After PBS washing, the samples were subjected to incubation with secondary antibody (Alexa Fluor 488/594, 1:200-1:500) for 1 hour at room temperature. Nuclei were counterstained with DAPI, finally captured by Olympus BX63 microscope (Olympus, Japan).

**RNA sequencing and analysis**

Stranded mRNA libraries were prepared using the ​​Illumina® Stranded mRNA Prep Kit. Paired-end sequencing (2 × 150 bp) was performed on the ​​Illumina NovaSeq 6000​​ platform. Gene expression was quantified as ​​TPM (Transcripts Per Million)​​ using ​​RSEM. Differential expression analysis was conducted with ​​DESeq2​​ (v1.46), applying thresholds of |log2FoldChange| ≥1 and adjusted P <0.05. Data visualization included hierarchical clustering (​​pheatmap​​), and volcano plots (​​ggplot2​​) to assess sample variance and differentially expressed gene (DEG) patterns. Functional annotation of DEGs was performed via ​​cluster Profiler​​ (v4.14.4), including ​​KEGG pathway​​ and GO​ enrichment analyses, with ​​GSEA​​ applied to hallmark pathways to elucidate biological significance.

**Western blot**

Protein samples were extracted using RIPA lysis buffer supplemented with protease and phosphatase inhibitors. Protein concentrations were determined via BCA assay. 30 μg proteins were separated by SDS-PAGE and transferred to PVDF membranes. Membranes were blocked with 5% non-fat milk in Tris-buffered saline with 0.1% Tween-20 (TBST) for 1 hour at room temperature, then incubated with primary antibodies overnight at 4°C. Next, the membranes were washed with TBST followed by probing with HRP-conjugated secondary antibodies (anti-mouse/rabbit, 1:5000) for 1 hour. Protein bands were visualized using ECL substrate and imaged with ChemiDoc systems. Band intensities were quantified using Image J software, with GAPDH serving as a loading control. The primary antibodies used in this experiment including β-catenin (Abcam, Cat#ab32572, 1:1000), p-GSK3beta (Ser9) (Cell Signaling Technology, Cat#5558S, 1:1000), GSK3β (Cell Signaling Technology, Cat#12456T, 1:1000), AKT (Cell Signaling Technology, Cat#4691, 1:1000), and p-AKT (Cell Signaling Technology, Cat#4060, 1:1000). All experiments were repeated at least three times independently.

**Molecular docking analysis**

AutoDock Vina 1.1.2 software was utilized to molecular docking analysis[4] with GSK3β (PDB: P49841) as receptors and rutin (PubChem CID 5280805) as ligands. PyMOL software 2.6.2 was used to visualize the results.

**Molecular dynamic simulations**

Molecular dynamic simulations were performed for 100 ns using ​​GROMACS 2022.​​ The protein was modeled using the AMBER ff14SB force field, and the small molecule was described with the General AMBER Force Field (GAFF). A solvent box was created and filled with TIP3P water molecules. Simulations employed a 2 fs integration timestep. The binding free energy was calculated via MMPBSA​​ method according to the equation: *ΔGbind=ΔE_vdw_+ΔE_ele_+ΔE_pol_+ΔE_nonpol_-TΔS. The stability of the peptides was assessed through root mean square deviation (RMSD) and radius of gyration (Rg), which were subsequently used to construct the free energy landscape (FEL).

**Surface plasmon resonance (SPR)**

SPR was used to examine the binding affinity between rutin and GSK3β using Biacore T200 (Cytiva Sweden). Recombinant GSK3β protein was purchased from sinobiological (Protein Accession: 10044-H07B). GSK3β protein was immobilized on the surface of CM5 sensor chip. During the binding cycle, rutin passed over the chip at a flow rate of 30 μL/min for 90 s. Kinetic parameters were analyzed using Biacore Insight Evaluation Software.

**Cellular thermal shift assay (CETSA )**

CETSA was employed to assess the thermal stability of proteins upon ligand binding. DPCs were treated with or without rutin for 24 hours, harvested, and resuspended in ice-cold lysis buffer containing PMSF. The suspensions were divided into equal aliquots, followed by incubation at designated temperature (37°C, 41°C, 45°C, 49°C, 53°C, 57°C, and 61°C) for 10 minutes to induce protein denaturation. Next, the samples were centrifuged at 12,000 × g, and the resultant supernatants were analyzed by western blot to determine the expression of GSK3β.

**Statistical analysis**

The results were presented as mean ± SD from at least three independent experiments. Statistical significance was evaluated using one-way ANOVA, two-way ANOVA or Student’s t-test. Values of p < 0.05 were regarded as significant differences.

## Supplementary figure legends

**Figure S1** Rutin activates DPCs. (A) Chemical structure of rutin. (B) CCK8 analysis of cell viability of DPCs after exposure to various concentrations of rutin for 24 hours (n=4 One-way ANOVA, followed by Tukey’s multiple comparisons test). (C) Ki67 immunofluorescence staining showing cell proliferation of DPCs after exposure to rutin (40 μM) or minoxidil (10 μM) for 24 hours (n=3, one‐way ANOVA, followed by Tukey’s multiple comparisons test. (D) Scratch assay analysis of cell migration of DPCs after treatment with rutin (n=4, unpaired Student’s t‐test). (E-H) RT-qPCR analysis of hair growth associated genes in DPCs after exposure to rutin (40 μM) or minoxidil (10 μM) for 24 hours (n=3, one‐way ANOVA, followed by Tukey’s multiple comparisons test). Data are mean ± SD. **p* < 0.05, ***p* < 0.01 and ****p* < 0.001.

**Figure S2** (A) The isolation of single hair follicles from human occipital scalp region. (B) Representative images showing hair follicle morphology in anagen and catagen, showing DPCs detachment from the hair matrix during catagen.

**Figure S3** Rutin promotes hair growth *in vivo*. (A) Transverse images of H&E staining of mouse skin on day 15. (A, right) Quantification of skin thickness and number of HFs in transverse sections ( n=6, one‐way ANOVA, followed by Tukey's multiple comparisons test). Scale bar= 200 μm. (B) Ki67 immunofluorescence staining showing cell proliferation of mouse skin on day 15.**p* < 0.05, ****p* < 0.001.

**Figure S4** Rutin promotes the expression of genes involved in hair growth *in vivo*. RT-qPCR analysis of *Versican*, *Fgf7*, *Vegfa* mRNA expression in mouse skin on day 15 post-administration (n=5, one‐way ANOVA, followed by Tukey’s multiple comparisons test). Data are mean ± SD. ***p* < 0.01 and ****p* < 0.001.

**Figure S5** Transcriptomic analysis of the gene profiling of skin tissues after rutin treatment. (A) The number of DEGs of RNA sequencing results of mouse skin on day 15 (n=3). (B) Volcano plot illustrating the expression patterns of DEGs for mouse skin after topical administration of rutin (10 mg/mL). (C) GSEA illustrating Wnt signaling pathway enriched in mouse skin.

**Figure S6** Higher-magnification views of Figure 1G.

**Figure S7** Rutin activates β-catenin signal *in vitro*. (A) Western blot analysis of β-catenin protein level in DPCs after exposure to rutin (40 μM) for indicated time. n=5. (B) β-catenin immunofluorescence staining and (C-E) RT-qPCR analysis of *CCND1*, *AXIN2*, and *LEF1* genes in DPCs following treatment with rutin (10, 20, 40 μM) for 24 hours (n=3, one‐way ANOVA, followed by Tukey’s multiple comparisons test). Data are mean ± SD. **p* < 0.05, ***p* < 0.01 and ****p* < 0.001.

**Figure S8** Molecular docking analysis of rutin/β-catenin complex.

**Figure S9** Molecular dynamic simulations analysis of rutin/GSK3β complex. (A-C) Molecular dynamics simulation illustrating the stability binding of rutin and GSK3β via Rg (A), RMSF (B), and energy (C).

**Figure S10** AKT is not required for GSK3b phosphorylation. (A) Molecular interactions between rutin and AKT (A). (B-D) Western blot analysis of AKT, p-AKT, GSK3β, p-GSK3β and β-catenin protein levels in DPCs with or without AKT inhibitor MK-2206 treatment in the presence of rutin for 24 hours. n=5 for B and C. n=3 and n=4 for D. Data are mean ± SD. **p* < 0.05, ***p* < 0.01 and ****p* < 0.001, one‐way ANOVA, followed by Tukey’s multiple comparisons test.

**Figure S11** GSK3β is essential for rutin functions in DPCs. (A) Western blot analysis to detect GSK3β protein expression after transfected with GSK3β siRNAs. (B) CCK-8 analysis of DPC proliferation in the presence or absence of rutin treatment after GSK3β knockdown (n=4). (C) Western blot analysis of β-catenin protein EXPRESSION in DPCs in the presence or absence of rutin treatment after GSK3β knockdown (n=5). (D) RT-qPCR analysis of *CCND1* mRNA level in DPCs in the presence or absence of rutin treatment after GSK3β knockdown (n=3). Data are mean ± SD. **p* < 0.05, ****p* < 0.001, one‐way ANOVA, followed by Tukey’s multiple comparisons test.

**Supplementary Figures**

**Figure S1**


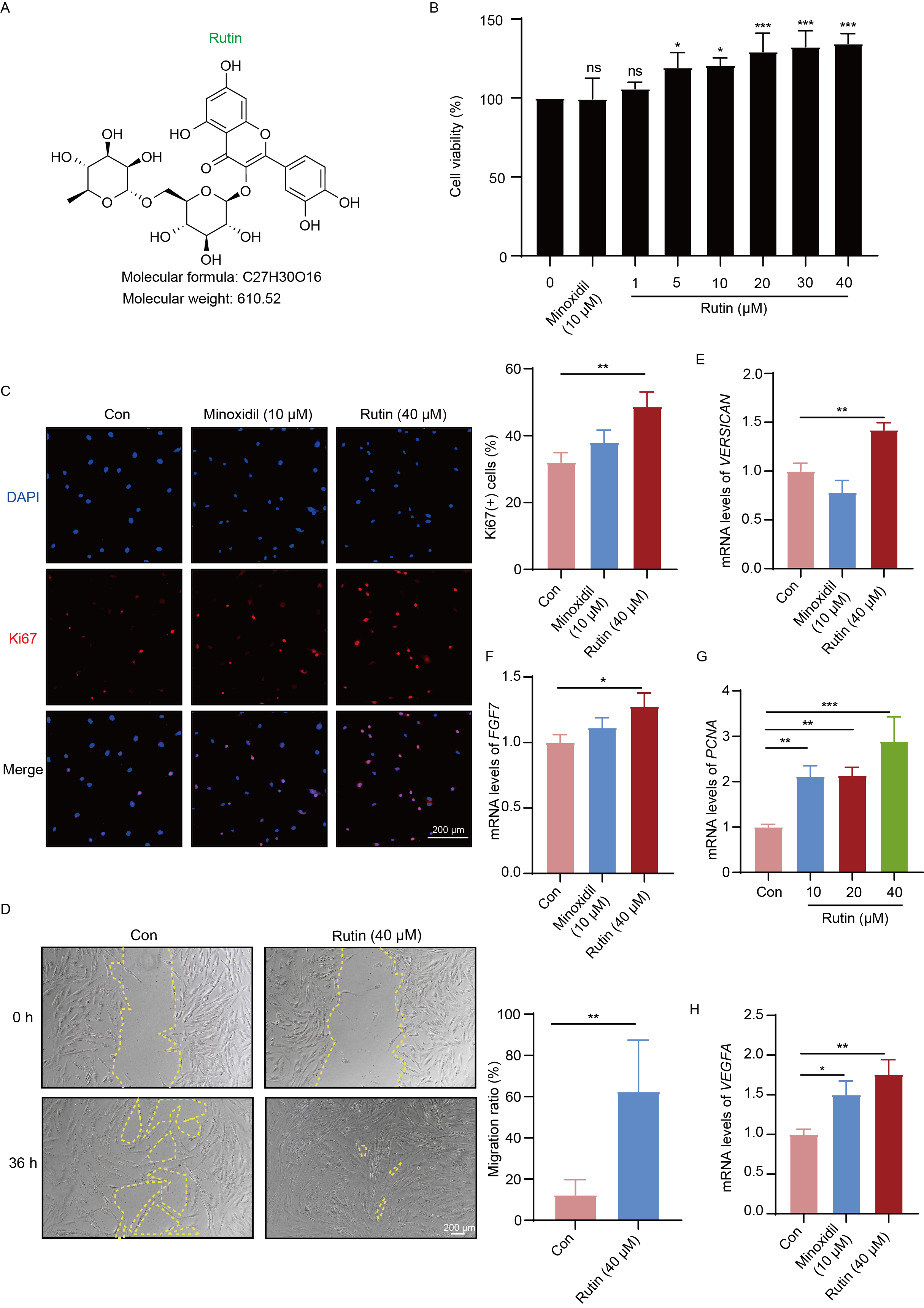
**Figure S2**

**
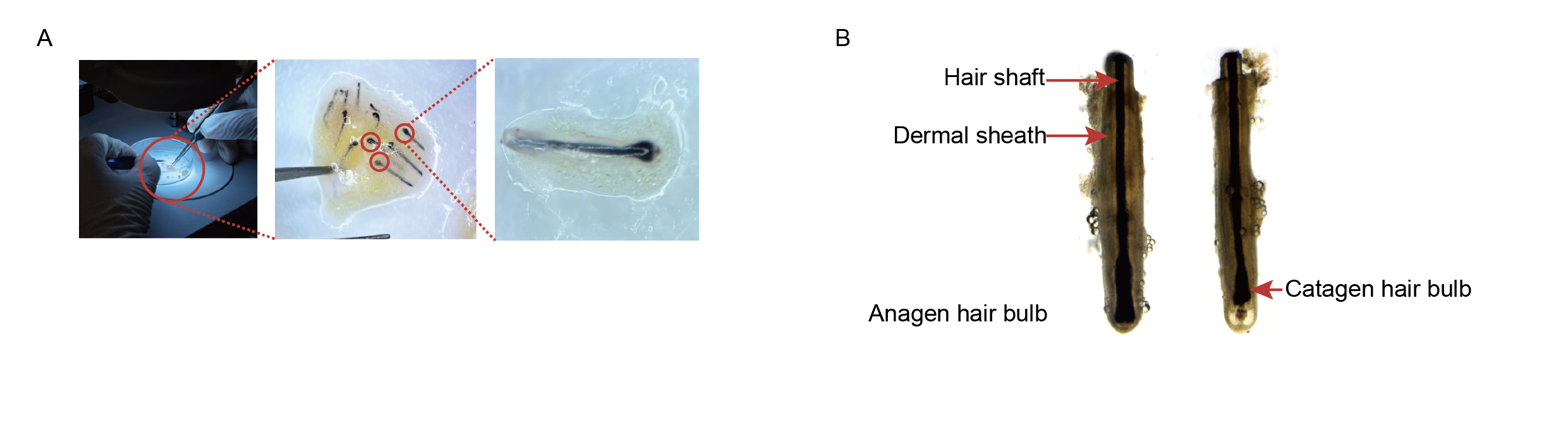
**

**Figure S3**

**
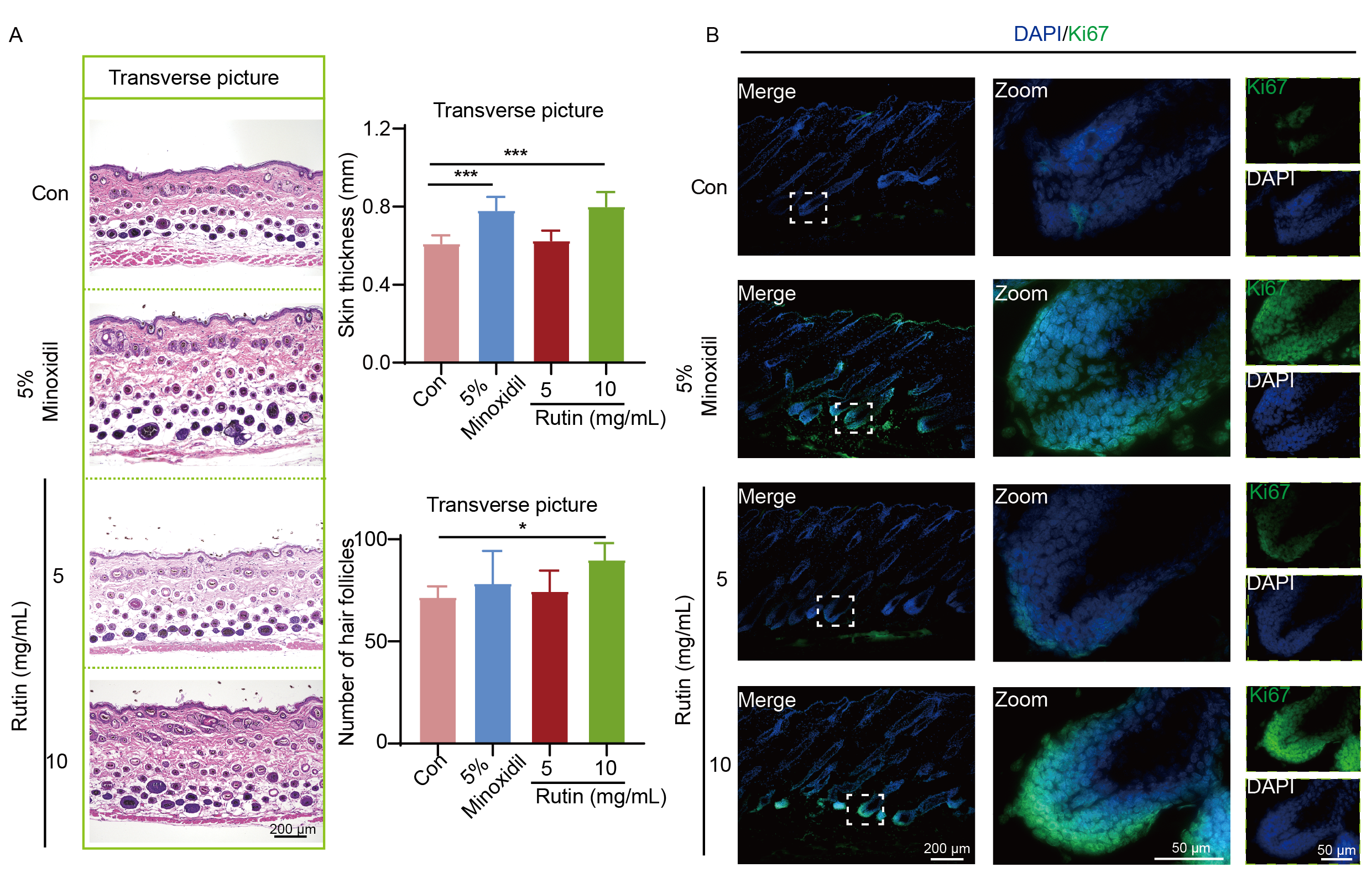
**

**Figure S4**

**
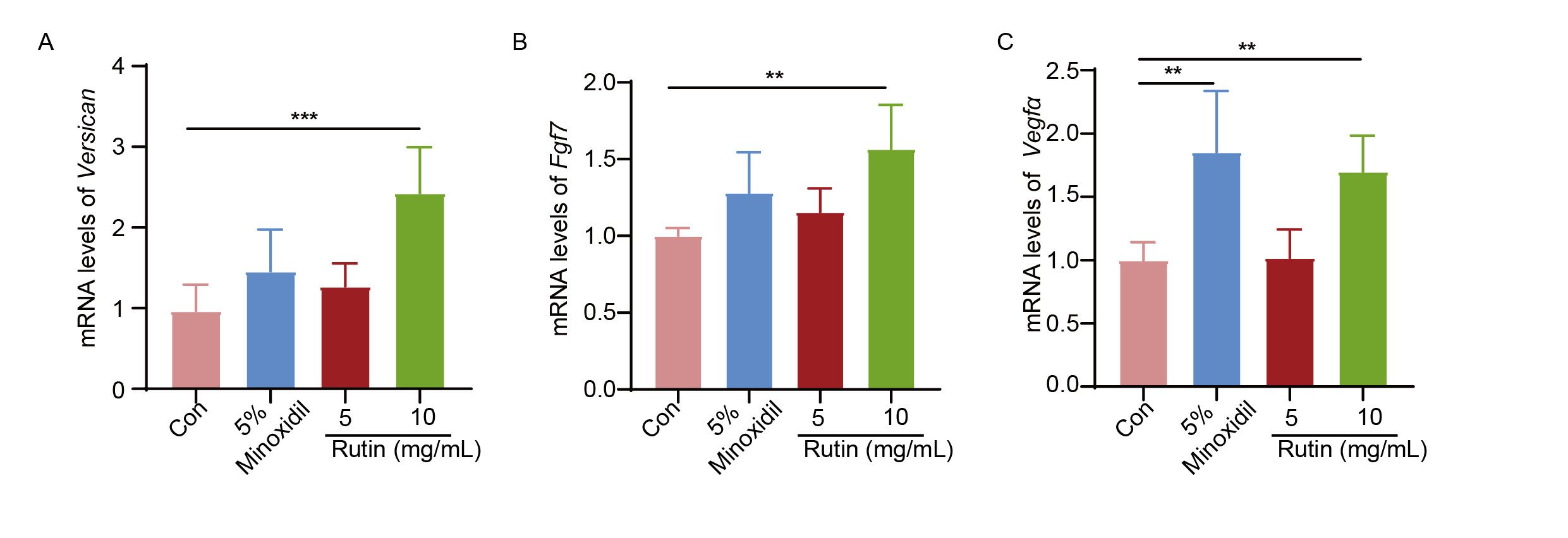
**

**Figure S5**

**
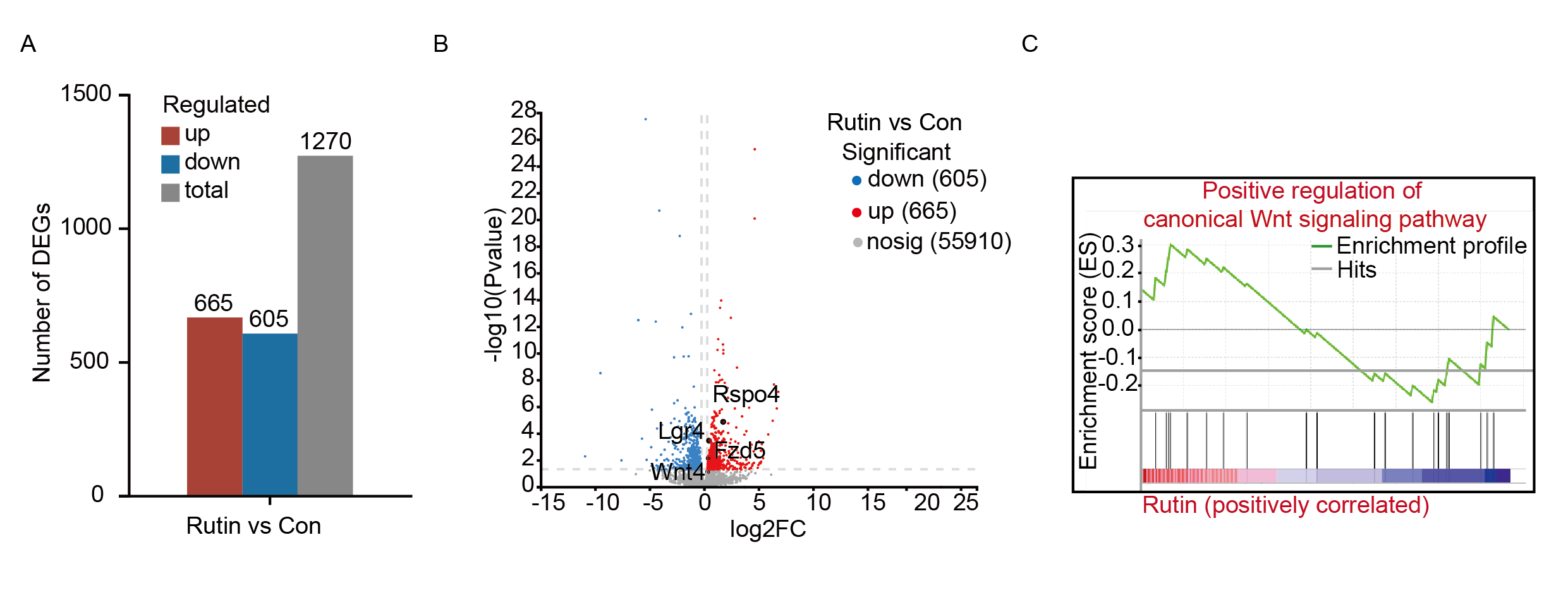
**

**Figure S6**

**
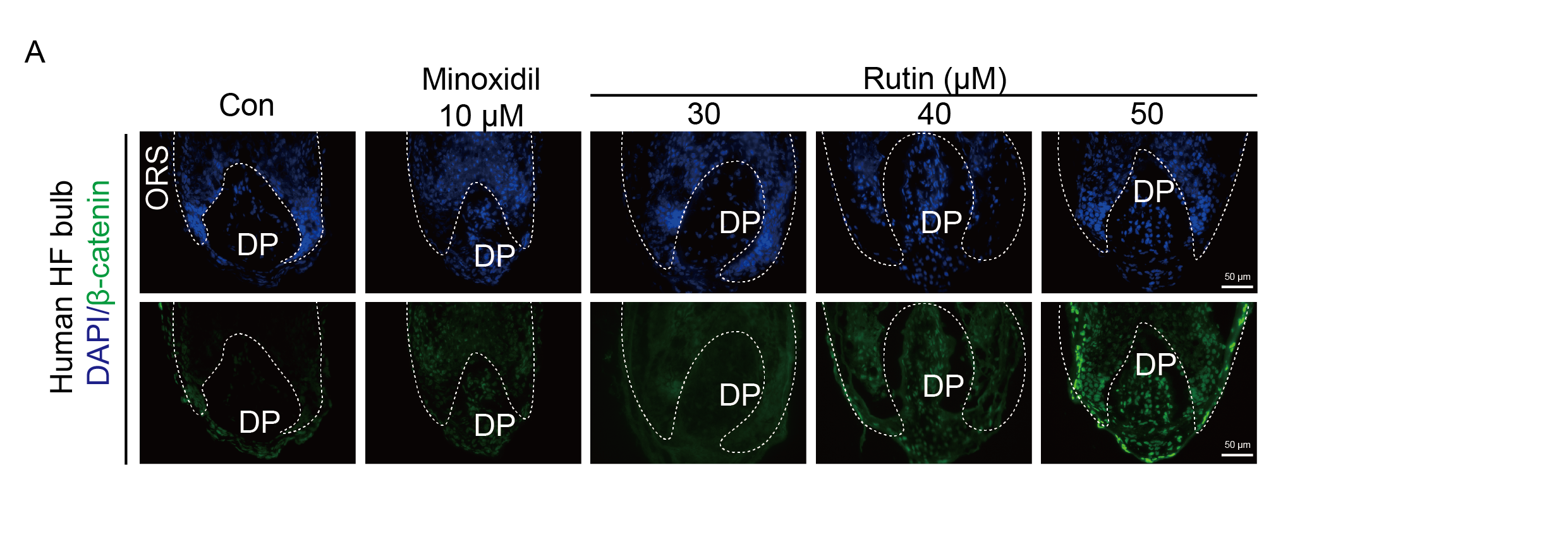
**

**Figure S7**

**
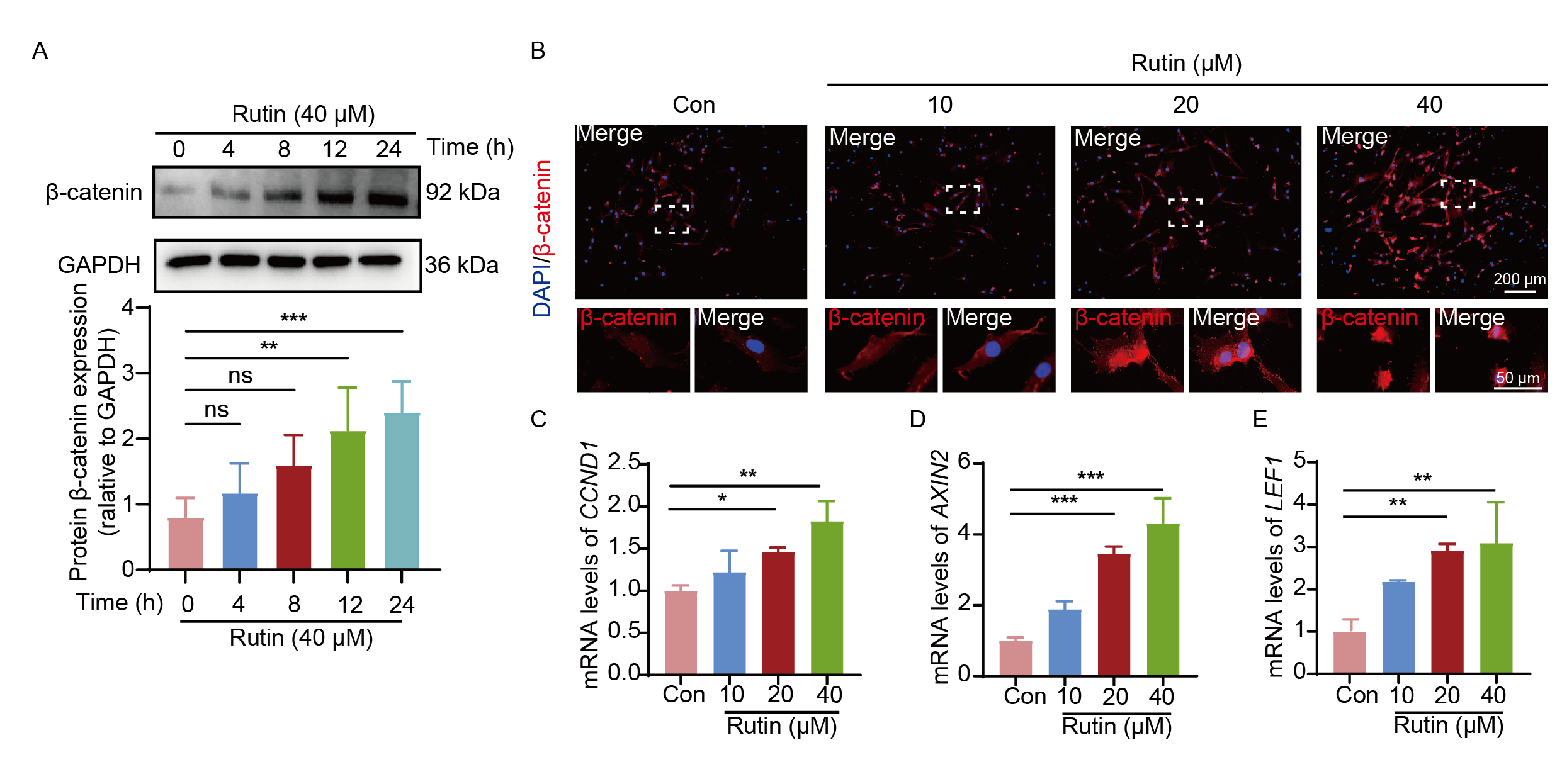
**

**Figure S8**

**
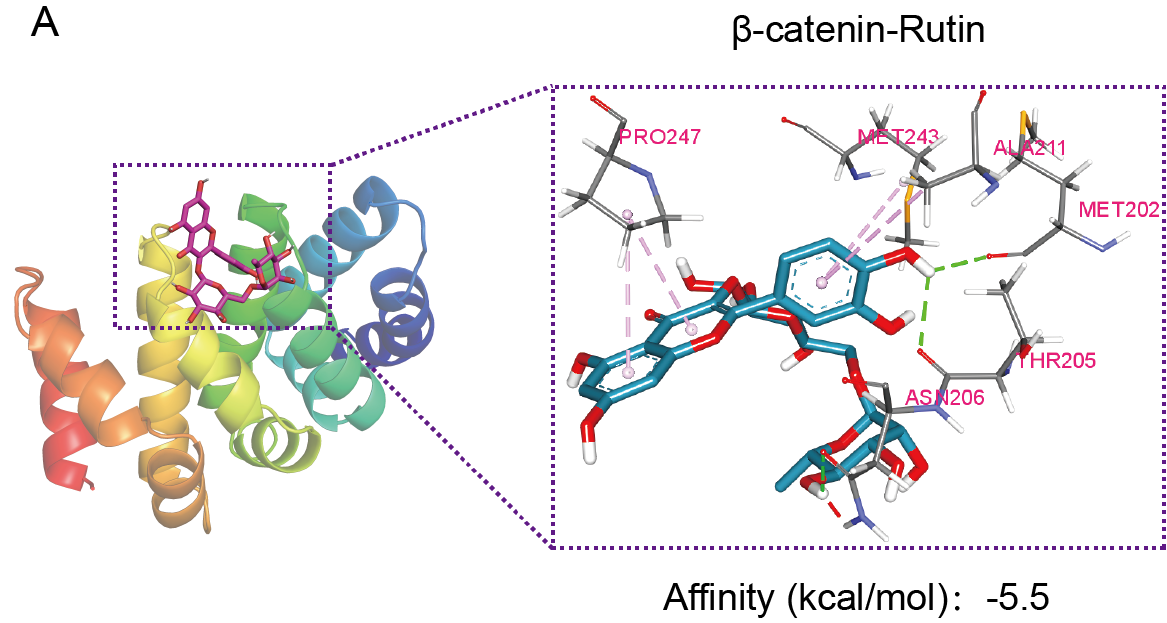
**

**Figure S9**

**
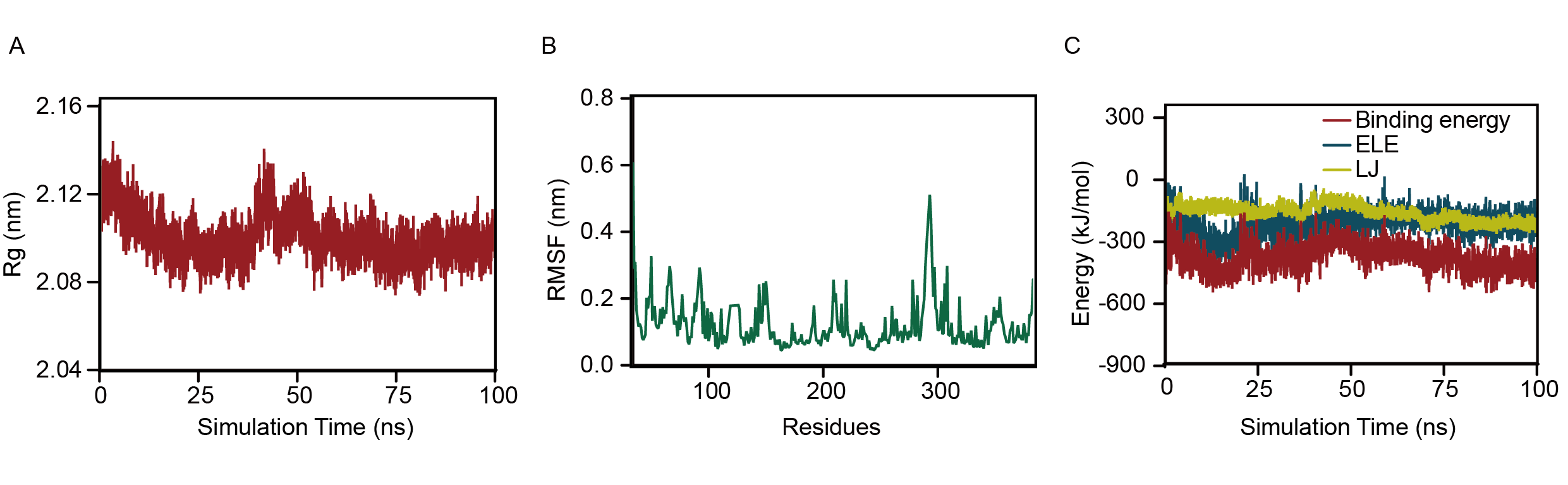
**

**Figure S10**

**
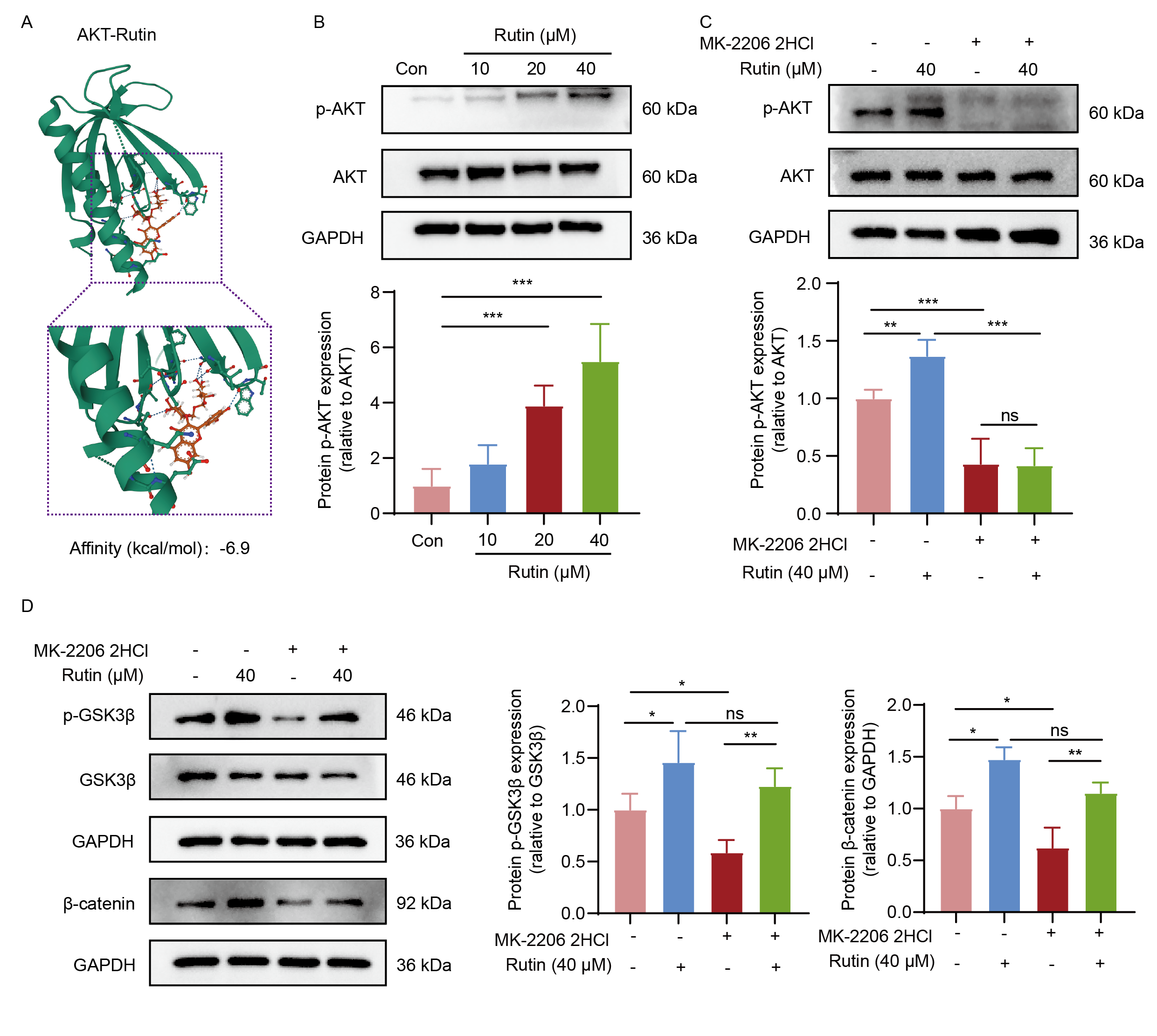
**

**Figure S11**


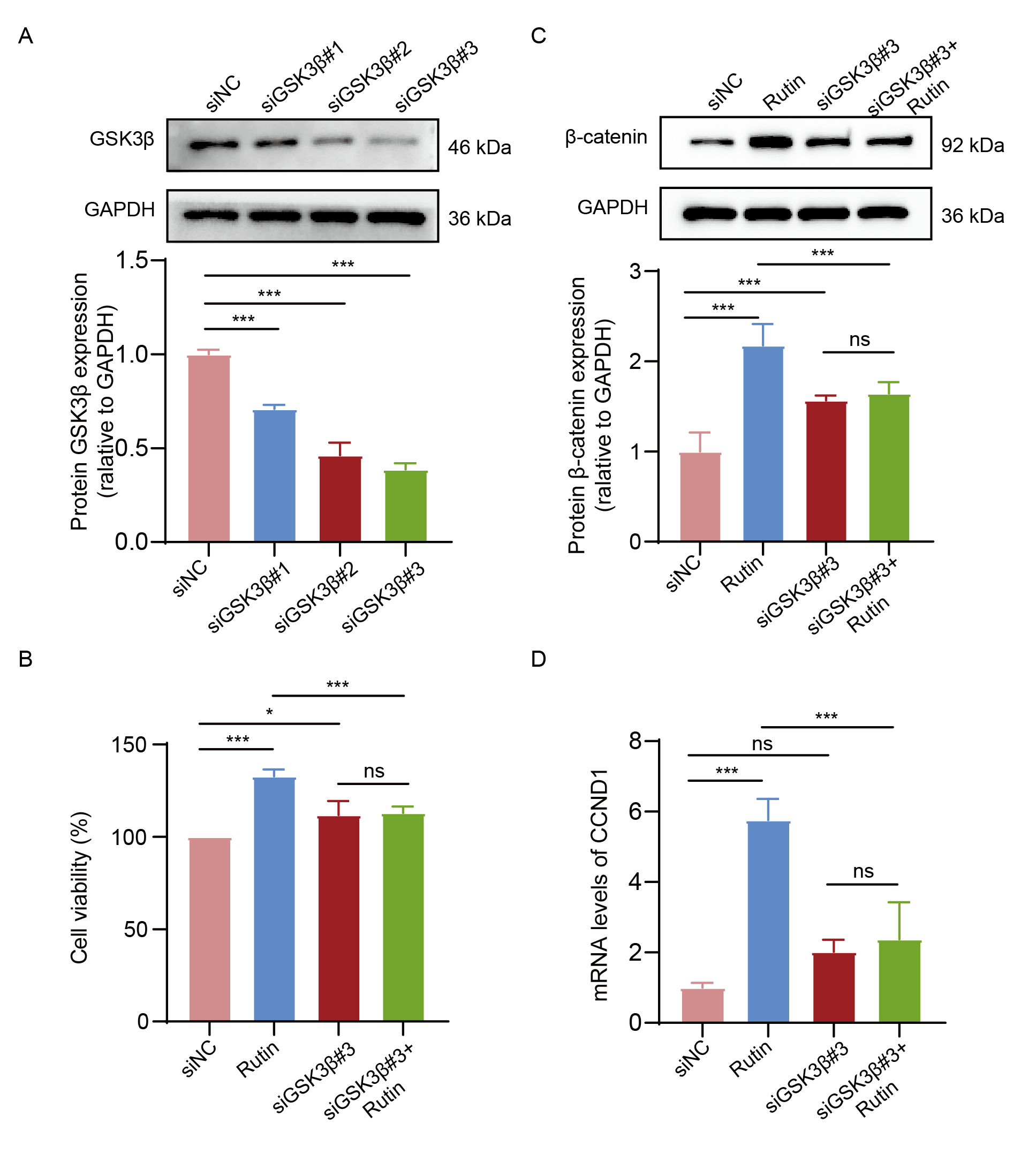


**Table S1** Binding energy and composition in a stable state (unit: kJ/mol)

| Complex | ΔE_vdw_ | Δ_Eele_ | ΔE_pol_ | ΔE_nonpol_ | ΔE_MMPBSA_ | TΔS | *ΔGbind |
| --- | --- | --- | --- | --- | --- | --- | --- |
| Protein/Ligand | -237.054±3.251 | -51.016±1.466 | -236.43±1.982 | -29.716±0.12 | -81.355±2.825 | -22.725±0.385 | -58.631±3.165 |

## References

[1] K. Cheng, J. Lin, M. Wu, J. Wang, X. Liu, K. Yang, C. Ni, Q. Liu, J. Wu, W. Wu, Berberine promotes hair growth by targeting Axin2 and activating Wnt/β-catenin pathway, Phytomedicine : international journal of phytotherapy and phytopharmacology 141 (2025) 156669.

[2] M. Zhu, Y. Fang, Y. Huang, W. Qiu, L. Ning, Y. Li, C. Zhu, X. Song, Y. Wu, W. Zou, A. Wang, Y. Lu, Transcriptomics sequencing reveals Qu-shi-yu-fa Decoction promotes hair cycle and keratinization by upregulating FOXN1 and TGM3 to treat androgenetic alopecia, Phytomedicine : international journal of phytotherapy and phytopharmacology 143 (2025) 156837.

[3] J. Wang, X. Li, X. Zhao, S. Yuan, H. Dou, T. Cheng, T. Huang, Z. Lv, Y. Tu, Y. Shi, X. Ding, Lactobacillus rhamnosus GG-derived extracellular vesicles promote wound healing via miR-21-5p-mediated re-epithelization and angiogenesis, Journal of nanobiotechnology 22(1) (2024) 644.

[4] J.Q. Hu, C.Q. Ning, F.C. Pi, X.N. Cai, J. Zhou, N. Wang, L.L. Yu, H. Zhou, Y. Xie, Suppressing ferroptosis via modulating FTH1 by silybin for treatment of renal fibrosis, Phytomedicine : international journal of phytotherapy and phytopharmacology 145 (2025) 156937.
